# Comparative performance of PROMIS Sleep Disturbance computerized adaptive testing algorithms and static short form in postmenopausal women

**DOI:** 10.1186/s41687-025-00849-6

**Published:** 2025-02-17

**Authors:** Andrew Trigg, Claudia Haberland, Huda Shalhoub, Christoph Gerlinger, Christian Seitz

**Affiliations:** 1 Clinical Statistics and Analytics, Bayer plc, Reading, UK; 2 Market Access, Bayer AG, Berlin, Germany; 3 Clinical Customer Centricity, Bayer AG, Berlin, Germany; 4 Clinical Statistics and Analytics, Bayer AG, Berlin, Germany; 5 Gynecology, Obstetrics and Reproductive Medicine, University Medical School of Saarland, Homburg, Saar, Germany; 6 Clinical Development and Operations, Bayer AG, Berlin, Germany; 7 Institute of Clinical Pharmacology and Toxicology, Charité, Berlin, Germany

## Abstract

**Background:**

The Patient-Reported Outcomes Measurement Information System (PROMIS) Sleep Disturbance v1.0 item bank (27 items) measures sleep disturbances. Rather than the full item bank, an 8-item short form (PROMIS SD SF 8b) or computerized adaptive testing (CAT) can be used. This study compares the performance of the PROMIS SD SF 8b with two CAT algorithms in postmenopausal women.

**Methods:**

This is a secondary analysis of data collected for the original psychometric testing of the PROMIS Sleep Disturbance item bank, in a sub-sample of women aged ≥55. A graded response model (GRM) was fitted for the item bank, then simulations evaluated the performance of CAT algorithms and the short form, in terms of root mean square error (RMSE) versus the latent trait estimate derived from the full bank. Two CAT algorithms were tested: CAT1 (stop once standard error <0.3 or 12 items administered) and CAT2 (stop once 8 items administered). Convergent and divergent hypotheses for validity were tested through correlations with the Pittsburgh Sleep Quality Index (PSQI) and Epworth Sleepiness Scale (ESS). Known-groups comparisons were made between those with and without self-reported sleep disorder.

**Results:**

A sample of 337 women was analyzed. Unidimensionality and item-level fit to the GRM was supported; however, the local independence assumption was violated. The CAT1 algorithm showed 4.18 items on average, with a minor decrease in performance (higher RMSE value) compared to CAT2 or the PROMIS SD SF 8b. Administering 8 items adaptively (CAT2) compared to fixed (PROMIS SD SF 8b) performed similarly (RMSE difference = 0.001). Reliability exceeded 0.90 across most of the latent trait for all approaches. Correlations with the PSQI and ESS were largely as hypothesized, with minor differences in coefficient values between the approaches (all within 0.05). Women reporting a sleep disorder had greater sleep disturbance than those who did not (*p* < 0.001 for all).

**Conclusions:**

The results of this study support using the PROMIS Sleep Disturbance item bank in postmenopausal women. The choice of PROMIS SD SF 8b versus CAT can largely be driven by practical reasons (respondent burden and operational complexity) rather than concerns of differential reliability and validity.

**Supplementary information:**

The online version contains supplementary material available at 10.1186/s41687-025-00849-6.

## Introduction

The menopausal transition reflects the natural decline of follicular estrogen production because of ovarian aging. Physiologically, this transition process spans over several years (peri-menopause) which is characterized by increased variability and decline in sex hormone levels and menstruation. Transition results in the postmenopausal state that is characterized with low estradiol levels and permanent absence of menstruation [1]. Sleep disturbances, recognized as a key domain of health-related quality of life (HRQL), are commonly experienced and reported by peri- and postmenopausal women [2–4]. In addition to short-term impacts such as fatigue and cognitive functioning, longer-term risks of sleep deprivation include obesity and diabetes [5]. Nighttime awakenings due to vasomotor symptoms (VMS; also known as hot flashes) contribute to sleep disturbances associated with menopause, but they are not the only factor involved [6, 7].

The Patient-Reported Outcomes Measurement Information System (PROMIS) Sleep Disturbance v1.0 item bank is a collection of 27 self-reported questions measuring frequency and intensity of different aspects of sleep disturbances. The item bank was developed through an iterative process of literature searches, collecting and sorting items, expert content review, qualitative patient research and quantitative testing [8]. An important factor in the quantitative testing is the use of item response theory (IRT) to select appropriate items and develop scoring. IRT methods facilitate the use of short forms or computerized adaptive tests (CATs), where precise estimates of sleep disturbance can be obtained with a subset of the 27 items in the full bank [9, 10]. IRT methods also provide estimates of the amount of information each item provides across the continuum of sleep disturbance severity, where some items are most informative at lower severity levels and vice versa [11].

An 8-item short form has been developed based on selecting the 8 best-performing items of the full PROMIS sleep disturbance 27 item bank referred to as the PROMIS SD SF 8b [10]. Performance was evaluated in a mixed sample from the US general population and sleep clinics, across a range of criteria including discrimination, raw score mean, percentage of times selected in CAT simulations and expected information [10]. The PROMIS SD SF 8b has been tested in cognitive interviews with postmenopausal women experiencing VMS, where it was deemed relevant and easily understood [12]. In addition, it has been administered in clinical trials assessing treatment of VMS in postmenopausal women, including the series of OASIS studies assessing elinzanetant [13–16]. The PROMIS SD SF 8b is static in nature, in the sense that all respondents see the same fixed set of 8 items. In contrast, CAT administration provides individualized assessments where, based on responses to preceding items, an algorithm selects the most informative item to administer next [9, 17, 18]. Subsequent items are administered until a predefined stopping criterion is met, such as a minimum acceptable precision in terms of standard error (SE), or a maximum number of items. Advantages of CAT include increased relevance to individual respondents, and the possibility to achieve equal or even greater precision with fewer items, reducing the burden of participants in a clinical trial [18].

The aim of this study is to compare the performance of the static PROMIS SD SF 8b with CAT algorithms in a sample of postmenopausal women. Ultimately, this might inform an alternative and improved way to measure postmenopausal sleep disturbance in future studies using the PROMIS Sleep Disturbance item bank.

## Methods

### Data source

This is a secondary analysis of data collected for the original psychometric testing of the PROMIS sleep disturbance item bank and short form creation, as described in further detail by Buysse et al. [8] and Yu et al. (2011) [10]. In brief, the dataset comprises data from 1993 adults from the general US population (734 of which had a self-reported sleep disorder such as insomnia, sleep apnea or restless leg syndrome), plus a separate clinical sample of 259 patients with sleep disorders recruited from the University of Pittsburgh Medical Center. This yields a pooled sample of 2252 participants. The data is available for download from the HealthMeasures Dataverse (10.7910/DVN/XESLRZ).

A subset of the pooled sample was used for this analysis to focus on postmenopausal women. The dataset does not include information on menopause, so all women aged 55 or above were considered eligible for analysis (given 90% of those with a natural menopause are postmenopausal by this age) [19]. This choice of age cut-off is in line with previous research [20], and will provide specificity. However, some women with earlier natural menopause will have been missed. Therefore, a sensitivity analysis of the PROMIS SD SF 8b versus CAT performance was performed based on women aged 49 or above, the average age of natural menopause [19]. An additional sensitivity analysis was conducted based on women aged 40–65, given this reflects an inclusion criterion for the previously mentioned OASIS studies of elinzanetant [15, 16].

### Measures

Demographic variables, global health ratings, and self-reported sleep problems were collected as described by Buysse et al. [8] and Yu et al. (2011) [10]. The full PROMIS sleep disturbance item bank comprises 27 items where respondents rate sleep problems ‘in the past 7 days’. Items are rated on 5-point scales, where some are reverse-coded to ensure higher scores always indicate greater sleep disturbance. The English language version was used for all participants, who completed the items in computerized format. The PROMIS sleep disturbance item bank, and the PROMIS SD SF 8b, can be downloaded from https://www.healthmeasures.net/search-view-measures.

The Pittsburgh Sleep Quality Index (PSQI) measures patient-reported sleep quality and disturbances over a 1-month period [21]. Responses to 19 items generate seven ‘component’ scores, which are summed to yield a ‘global’ score ranging from 0 (good sleep quality) to 21 (poor sleep quality). However, formatting issues with data for PSQI Item 1 and 3 (usual bed time and getting up time) prevented our accurate calculation of the ‘Habitual sleep efficiency’ component score, so only the other six components are presented further (see Supplementary Appendix for further details). The Epworth Sleepiness Scale (ESS) measures daytime sleepiness using 8 items, each scored 0–3, that are summed to yield a score ranging from 0 (no propensity for dozing during daytime activities) to 24 (high propensity for dozing during daytime activities) [22, 23]. ESS scores >10 represent excessive daytime sleepiness [24]. The data source also included a PROMIS ‘sleep-related impairment’ item bank which is not the focus of this study.

### Statistical methods

Analyses were based on IRT modelling, namely a unidimensional graded response model (GRM) with a logistic link function. In this model, the probability of selecting each item response is conditional on a continuous latent trait representing the continuum of sleep disturbance, plus item-specific parameters. The latent trait is assumed normally distributed with mean = 0 and standard deviation = 1, where respondents’ location on the trait represents the extent of their sleep disturbance (0 = average sleep disturbance in the sample, 2 = 2 standard deviations above the average). The item-specific parameters in the unidimensional GRM are discrimination and difficulty. Discrimination parameters (one for each item) characterize the strength of the relationship between the latent trait and response probabilities, where higher values indicate that an item’s responses differentiate well across the latent trait (values >1 deemed supportive of an item’s performance as per [8]). Difficulty parameters (4 for each item, b1-4) represent the thresholds between cumulative response probabilities, where for example b2 = 0.8 means that the probabilities of responding in categories 1/2 versus 3/4/5 are equal at a location of 0.8 on the latent trait. In this way, both respondents and items are represented on the same metric of the latent trait. An additional aspect of IRT, important to CAT, is that each item has an information function showing its ability to discriminate respondents at different locations on the trait, where this is driven by the item parameters. Information is related to reliability where the specific formula relating the two varies according to how the latent trait is estimated [17, 25]. The unidimensional GRM was estimated using marginal maximum likelihood [26, 27], with the trait location estimated using Bayes modal estimation (BME). When using BME to estimate trait locations, specifying a standard normal prior distribution (i.e. mean = 0 and standard deviation = 1), reliability = 1-(1/[1 + information]) [17].

Three core assumptions underly IRT models, namely dimensionality, local independence, and functional form [28]. Unidimensionality was assessed by plotting the eigenvalues of the polychoric correlation matrix of items in the bank, referred to as a scree plot [29, 30]. Only having one eigenvalue prior to the ‘elbow’ of the plot, and a ratio of the first to second eigenvalue exceeding 4:1, are deemed supportive of unidimensionality [8, 31]. Local independence is the assumption that item responses are unrelated after controlling for the latent trait, assessed by Yen’s Q3 indices for each item pair which is the Pearson correlation between residuals (values >0.5 proposed to indicate problematic local dependence in a PROMIS item bank [8, 31]). Functional form concerns the extent to which a unidimensional GRM with a logistic link function fits the data, assessed by S–X^2^ values for each item which represent the magnitude of differences between observed and predicted item responses [32]. Given the 27 items, the S–X^2^ p values were corrected for multiplicity using a Benjamini-Hochberg adjustment (*p* < 0.05 after adjustment indicates item misfit) [11, 33].

Simulations evaluated the performance of CAT algorithms based on the estimates of the latent trait and item parameters derived from the data. The item parameters were estimated based on the full item bank and fixed at these values. These were post-hoc simulations in the sense that the observed item responses in the dataset were used. CAT algorithms comprise 4 steps [17]: (1) *starting step* where the first item (or set of items) is selected; (2) *test step* where additional items are iteratively selected and trait location is re-computed after each additional response; (3) *stopping step* which defines the rules to stop administering additional items; (4) *final step* which provides the final estimate of trait location.

For the starting step, the item providing maximum Fisher information (MFI) at a trait location of zero was selected. In the test step, additional items were selected based on MFI at the provisionally estimated trait location (BME with standard normal prior distribution). BME was chosen as it can estimate trait locations when all item responses are at the minimum or maximum, a common occurrence in the early iterations of CAT algorithms. For the stopping step, two different rules were applied, subsequently referred to as CAT1 and CAT2. In CAT1, the algorithm stopped when either SE < 0.3 (≈ reliability >0.9) or 12 items had been administered, which reflects standard practice for PROMIS [34]. In CAT2, the algorithm stopped once 8 items had been administered, representing an adaptive alternative to the PROMIS SD SF 8b with same length. In the final step, trait location and associated SE was estimated using BME. To assess sensitivity to the choice of starting step, an alternate approach was to randomly select one of the five items providing MFI between a trait location of −2 and 2 (referred to as a ‘randomesque’ procedure in the CAT literature) [17].

The estimated latent trait locations using the full item bank were treated as the ‘true’ estimates, where estimates from CAT1, CAT2 and the short form were compared to this in terms of Pearson correlation and root mean square error (RMSE). In addition, the final estimated SEs were presented by decile of the true latent trait. The relative efficiency of each approach was also presented by decile of the true latent trait, where efficiency is defined as information divided by the number of items shown. The relative efficiency is then calculated as a ratio of the efficiencies of two approaches, e.g. for CAT1 versus CAT2, relative efficiency = efficiency_CAT1_/efficiency_CAT2_ where values above 1 indicate higher efficiency for CAT1, values of 1 indicate equal efficiency, and values below 1 indicate higher efficiency for CAT2.

To assess convergent/divergent evidence of validity for the various PROMIS sleep disturbance scores, correlations were calculated with the ESS Total Score (Spearman’s) and the different PSQI component scores (polyserial). Based on findings in the original short form development [10], correlations between 0.2 and 0.3 were expected with the ESS (i.e. divergent evidence; r = 0.25 was previously observed between the ESS Total Score and the full 27-item bank latent trait estimate). The original short form development study only reported correlations with the PSQI global score, so an alternate convergent evidence hypothesis was formulated where each calculable PSQI component was ranked from highest to lowest expected correlation based on item content as follows: Subjective sleep quality, Sleep latency, Sleep disturbances, Sleep duration, Use of sleeping medication, Daytime dysfunction. The hypothesis was tested by comparing the observed rankings of correlation coefficients to the expected rankings.

Known-groups evidence for validity was obtained by comparing the various PROMIS Sleep Disturbance trait estimates of women with a sleep disorder versus those without. Sleep disorder was self-reported within the general US population sample (as part of the same questionnaire as the PROMIS items) or clinician-diagnosed within the clinical sample. A two-sample t-test was conducted and Cohen’s d presented, interpreted with cut-offs for small (d = 0.2), moderate (d = 0.5) and large (d = 0.8) [35].

Analyses were conducted in R using the mirt, psych and catR packages [17, 27, 36]. R code for the main analyses is provided in the Supplementary Appendix.

## Results

The sample comprised 337 women, where 281 were recruited from the general US population (of which 88 had self-reported sleep problems) and 56 were recruited from the clinical sample all with reported sleep disorders. The women had a median age of 62 (range 55 to 85), where 90.8% were white, 7.4% black, 0.9% Native American or Alaskan, 0.6% Native Hawaiian or Pacific Islander and 0.3% Asian, with 3.3% Hispanic or Latino. Education attainment was high school or less (17.5%), some college (40.1%), college degree (20.5%), and advanced degree (22.0%). 19.0% had excessive daytime sleepiness (as per ESS score >10).

**Fig. 1:**
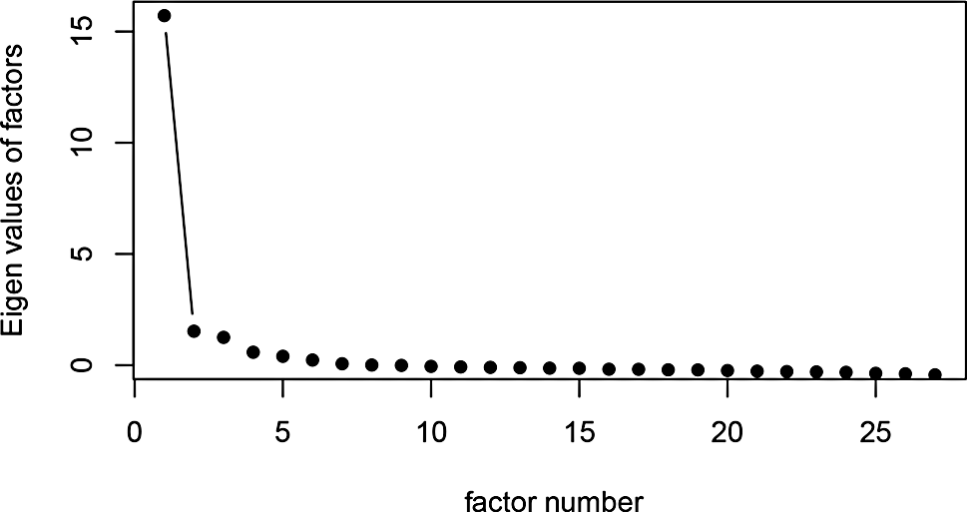
Scree plot

Unidimensionality of the item bank was supported, where only one factor was prior to the ‘elbow’ of the scree plot and the ratio of the first to second eigenvalue greatly exceeded 4:1 (Fig. 1). Local dependence (Yen’s Q3 >0.5) was identified for the following item pairs: S42 ‘It was easy for me to fall asleep’ & S44 ‘I had difficulty falling asleep’ (Q3 = 0.759); S115 ‘I was satisfied with my sleep’ & S116 ‘My sleep was refreshing’ (Q3 = 0.533); S68 ‘I felt worried at bedtime’ & S70 ‘I felt sad at bedtime’ (Q3 = 0.527); and S44 ‘I had difficulty falling asleep’ & S45 ‘I laid in bed for hours waiting to fall asleep’ (Q3 = 0.512). Of note, only the item pair S115 & S116 is present in the PROMIS SD SF 8b. No significant item misfit (assessed by S–X^2^ values) was identified, supporting the functional form assumption.

**Fig. 2:**
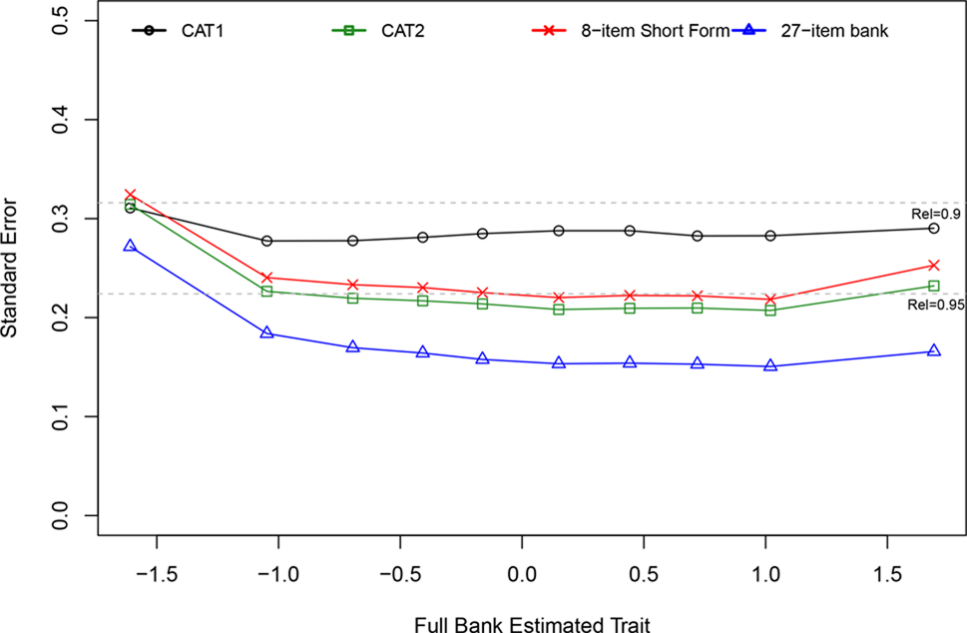
Standard error of different approaches, split by decile of latent trait

**Fig. 3:**
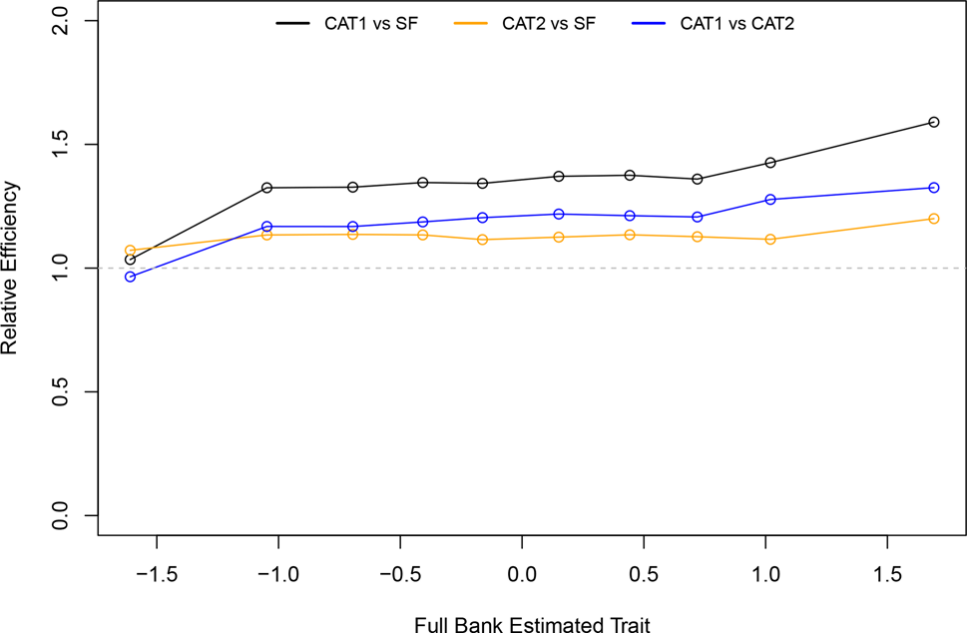
Relative efficiency of different approaches, split by decile of latent trait. Relative efficiency is the ratio of information per item. Values above 1 indicate higher efficiency for the approach listed first, values of 1 indicate equal efficiency, and values below 1 indicate higher efficiency for the approach listed second

Item discrimination parameters estimated by the GRM were all greater than 1 (range 1.18 to 4.17) and the difficulty thresholds were ordered from low to high as expected. Simulations assessed the performance of both CAT algorithms and the PROMIS SD SF 8b, compared to the full item bank (Table 1). Pearson correlations with the full item bank were consistently high, ranging from 0.96 to 0.97. The CAT1 algorithm showed only 3 or 4 items in most instances (86.1% of respondents), with a minor decrease in performance (higher RMSE value) compared to CAT2 or the PROMIS SD SF 8b with 8 items each. Administering 8 items adaptively (as per CAT2) compared to fixed (as per PROMIS SD SF 8b) performed similarly (RMSE difference = 0.001). The SEs of each approach show that all have high precision, with reliability exceeding 0.90 across most of the latent trait (Fig. 2). Relative efficiency, in terms of information per number of items administered, is displayed in Fig. 3. The CAT1 approach is notably more efficient than the PROMIS SD SF 8b, given it provides similar precision but with fewer items.

**Table 1:** CAT and short form performance versus the full bank

Approach	Correlation with full bank (Pearson)	RMSE	Mean number of items
CAT1 (SE < 0.3 or 12 items)	0.9605	0.2699	4.18
CAT2 (any 8 items)	0.9747	0.2176	8 (fixed number)
PROMIS SD SF 8b	0.9748	0.2166	8 (fixed number)

Exposure rates (i.e. the percentage of simulations each item is presented) are provided in the Supplementary Appendix. As several items were selected by the CAT algorithms over 90% of the time (and others missed entirely), a post-hoc sensitivity analysis was conducted where the maximum exposure rate was set at 80% (i.e. once an item had appeared in 80% of simulations, it was ineligible for further selection) [37]. This yielded similar results for CAT1 and CAT2 (correlation with full bank 0.9608 and 0.9733; RMSE 0.2693 and 0.2227), but some items were still consistently missed (see Supplementary Appendix for detailed results). Analyses based on a randomesque starting step did not affect the results (correlations 0.9638 for CAT1 and 0.9731 for CAT2) RMSE (0.2581 for CAT1, 0.2243 for CAT2; see Supplementary Appendix for detailed results).

Correlations with the ESS were all between 0.2 and 0.3 as hypothesized, where the different coefficients for each approach were all within 0.05 of each other (Table 2). Correlations with the PSQI component scores were generally in accordance with the expected rankings, where the top two highest coefficients (Subjective sleep quality and Sleep latency) were as hypothesized. The observed ranking of correlation coefficients for Sleep disturbances and Sleep duration was switched compared to expectations, although the differences in correlations were minor (0.05 or less). Daytime dysfunction was more highly correlated than Use of sleeping medication, contrary to expectations. The correlation coefficients with PSQI component scores were similar across each approach.

**Table 2:** Correlations with the PSQI component scores (polyserial) and ESS (Spearman’s)

	CAT1 (SE < 0.3 or 12 items)	CAT2 (any 8 items)	PROMIS SD SF 8b	Full item bank
**ESS total score**	0.2107 (0.1061–0.3107)	0.2496 (0.1465–0.3473)	0.2518 (0.1488–0.3494)	0.2056 (0.1008–0.3059)
PSQI*				
Component 1: Subjective sleep quality	0.8947 (0.8712–0.9142)	0.9196 (0.9013–0.9346)	0.9147 (0.8954–0.9306)	0.8923 (0.8682–0.9122)
Component 2: Sleep latency	0.7190 (0.6631–0.7669)	0.7112 (0.6541–0.7603)	0.7328 (0.6791–0.7787)	0.7860 (0.7414–0.8237)
Component 5: Sleep disturbances	0.6155 (0.5445–0.6778)	0.6330 (0.5643–0.6930)	0.6311 (0.5621–0.6913)	0.6520 (0.5860–0.7094)
Component 3: Sleep duration	0.6588 (0.5937–0.7153)	0.6781 (0.6159–0.7319)	0.6824 (0.6208–0.7356)	0.6550 (0.5894–0.7120)
Component 6: Use of sleeping medication	0.3767 (0.2812–0.4649)	0.3554 (0.2583–0.4453)	0.3455 (0.2478–0.4362)	0.3909 (0.2965–0.4778)
Component 7: Daytime dysfunction	0.4427 (0.3525–0.5247)	0.4758 (0.3887–0.5545)	0.4709 (0.3834–0.5501)	0.4940 (0.4087–0.5707)

*PSQI components are arranged in order of hypothesized correlation (highest at the top). Component 4 could not be calculated as described in methods

95% confidence intervals provided in parentheses

Women with sleep disorders had higher estimated trait values (indicating greater sleep disturbance) than those without (*p* < 0.001 for all approaches). Cohen’s d values were consistently large (CAT1, d = 0.97; CAT2, d = 1.02; PROMIS SD SF 8b, d = 1.00; full item bank, d = 0.97).

Sensitivity analysis was performed based on 484 women aged 49 or above. Results were highly comparable, where Pearson correlations with the full bank ranged from 0.96 to 0.97, RMSE ranged from 0.22 to 0.26, and all approaches were highly precise (reliability exceeding 0.90 across most of the latent trait, see Supplementary Appendix for detailed results).

Sensitivity analysis was also performed based on 590 women aged 40–65. Results were highly comparable, where Pearson correlations with the full bank ranged from 0.96 to 0.98, RMSE ranged from 0.17 to 0.26, and all approaches were highly precise (reliability exceeding 0.90 across most of the latent trait, see Supplementary Appendix for detailed results).

## Discussion

This study aimed to compare the performance of the static PROMIS SD SF 8b with two CAT algorithms, specifically within a sample of women in the postmenopausal age group (55+). The CAT1 algorithm is expected to yield a short questionnaire (3–4 items) for most respondents, with only a minor decrease in performance, so could be the preferred assessment tool if time for completion is an important issue (e.g. within clinical practice). The CAT2 algorithm (any 8 items) performed similarly to the static PROMIS SD SF 8b. This may reflect the fact that CAT performance (percentage of times each item was administered in simulations) was used to guide item inclusion within the short form [10]. It is important to consider the operational time and costs required to implement a CAT algorithm. Given CAT1 provides slightly lower precision and is associated with increased operational costs, while only reducing length by an average of four items, it could be argued that administering the static PROMIS SD SF 8b is preferable in future. An even stronger argument exists for preferring the PROMIS SD SF 8b over the CAT2 algorithm, as there is no practical gain in precision of adaptive testing to make the increased operational costs worthwhile.

Sensitivity analyses based on alternate age groups resulted in similar conclusions regarding the performance of the different approaches. In addition, modifying the CAT algorithms in terms of maximum exposure rates, or introducing a randomesque starting step, yielded similar results.

Evidence for validity in postmenopausal women was also obtained. Correlations with the PSQI and ESS were largely as hypothesized, with minor differences in coefficient values between the approaches. Known-groups evidence was also obtained, where women reporting a sleep disorder had consistently greater sleep disturbances than those who did not, across all three approaches. The findings from this study are largely comparable to those from the original PROMIS SD SF 8b development, where the authors saw a correlation between the PROMIS SD SF 8b and the full bank of 0.96, high reliability and similar correlations with ESS [10].

Notably, PROMIS measures are commonly expressed on a T-Score metric, where a score of 50 points represents the mean and 10 points represents a standard deviation, based on the general US population. However, this study used the latent trait metric (mean = 0 and standard deviation = 1) to align with the prior research and to avoid the assumption that the scores were calibrated in terms of the general population. The focus here is on postmenopausal women, where it is recommended that calibration is performed in the specific target population of interest [38, 39]. The difference in metric would not have any impact on the results of this study, comparing the performance of CAT algorithms with the PROMIS SD SF 8b.

Limitations of this study are acknowledged. The analyses in this study were reliant on IRT methods, which have an underlying assumption of local independence. This assumption was in fact violated for four item pairs in the full bank; therefore, it is possible that CAT algorithms that do not rely on IRT assumptions (namely tree-based methods) could perform better so could be evaluated in future work [40, 41]. Additionally, an assumption of being postmenopausal was made based on age, rather than being clinically confirmed. Women with early menopausal symptoms due to removal of ovaries or endocrine cancer treatment are likely to have been missed in this age-based sample. Issues with PSQI data formatting meant the global score and the ‘Habitual sleep efficiency’ component score could not be calculated. Finally, the US-based sample may limit the generalizability of results to non-US populations.

## Conclusions

Overall, the results of this study support the use of the PROMIS Sleep Disturbance item bank to measure this construct in postmenopausal women. Accordingly, this supports its continued use to assess efficacy endpoints of clinical trials in postmenopausal women. The choice of CAT algorithm versus the PROMIS SD SF 8b can largely be driven by practical reasons (respondent burden and operational complexity) rather than concerns of differential reliability and validity.

## Electronic supplementary material

Below is the link to the electronic supplementary material.

Supplementary Material 1

## Data Availability

The data is available for download from the HealthMeasures Dataverse (10.7910/DVN/XESLRZ). R code for the main analysis is provided in Supplementary Appendix.

## Abbreviations

BME: Bayes modal estimation
CAT: Computerized adaptive testing
ESS: Epworth Sleepiness Scale
GRM: Graded response model
HRQL: Health-related quality of life
IRT: Item response theory
MFI: Maximum Fisher information
PROMIS: Patient-Reported Outcomes Measurement Information System
PROMIS SD SF 8b: PROMIS Sleep Disturbance Short Form 8b
PSQI: Pittsburgh Sleep Quality Index
RMSE: Root mean square error
SE: Standard error
VMS: Vasomotor symptoms
