# Supplementary material for: Comparative performance of PROMIS Sleep Disturbance computerized adaptive testing algorithms and static short form in postmenopausal women
Source: J Patient Rep Outcomes. 2025 Feb 17;9:18. doi: 10.1186/s41687-025-00849-6 (PMC11832987; doi:10.1186/s41687-025-00849-6)
Supplement: Supplementary file 1 — Supplementary Material 1 [file 41687_2025_849_MOESM1_ESM.docx]

**Supplementary Appendix**

***Formatting issues with PSQI Item 1 and 3***

PSQI Item 1 (usual bed time) and Item 3 (usual getting up time) were designed to be recorded in military time (e.g. ‘2230’) as per the accompanying codebook for the dataset. However, the majority of observed values were not in a military time format, with example confusing values of ‘5’, ‘29’, ‘95’. For values such as ‘5’, it is unclear whether 17:00 or 05:00 is referred to. Additionally, the values for PSQI Item 1 and 3 were the same in several cases. A query was raised through the ‘contact owner’ function of the HealthMeasures Dataverse (<https://doi.org/10.7910/DVN/XESLRZ>) in an attempt to resolve this issue.

***Exposure rates***

| **Item** | **CAT1 exposure rate** | **CAT2 exposure rate** |
| --- | --- | --- |
| Sleep20 | 66.77% | 85.46% |
| Sleep42 | 8.01% | 8.01% |
| Sleep44 | 3.86% | 0.00% |
| Sleep45 | 0.30% | 0.30% |
| Sleep50 | 4.15% | 0.00% |
| Sleep65 | 0.00% | 0.00% |
| Sleep67 | 0.30% | 0.59% |
| Sleep68 | 0.30% | 0.00% |
| Sleep69 | 2.67% | 0.00% |
| Sleep70 | 0.00% | 0.00% |
| Sleep71 | 0.00% | 0.00% |
| Sleep72 | 0.89% | 3.56% |
| Sleep78 | 0.30% | 0.00% |
| Sleep86 | 0.30% | 1.19% |
| Sleep87 | 10.09% | 22.55% |
| Sleep90 | 87.83% | 92.88% |
| Sleep92 | 8.01% | 92.28% |
| Sleep93 | 0.30% | 0.00% |
| Sleep105 | 13.35% | 99.41% |
| Sleep106 | 0.00% | 0.00% |
| Sleep107 | 5.64% | 4.15% |
| Sleep108 | 1.48% | 5.04% |
| Sleep109 | 100.00% | 100.00% |
| Sleep110 | 12.76% | 93.47% |
| Sleep115 | 68.55% | 94.96% |
| Sleep116 | 21.66% | 96.14% |
| Sleep125 | 0.00% | 0.00% |

***Sensitivity Analysis - maximum exposure rate set at 80%***

CAT and Short Form performance versus the full bank

| **Approach** | **Correlation with full bank (Pearson)** | **RMSE** | **Mean number of items** |
| --- | --- | --- | --- |
| CAT1 (SE<0.3 or 12 items) | 0.9608 | 0.2693 | 4.37 |
| CAT2 (any 8 items) | 0.9733 | 0.2227 | 8 (fixed number) |
| PROMIS SD SF 8b | 0.9748 | 0.2166 | 8 (fixed number) |

Standard error of different approaches, split by decile of latent trait


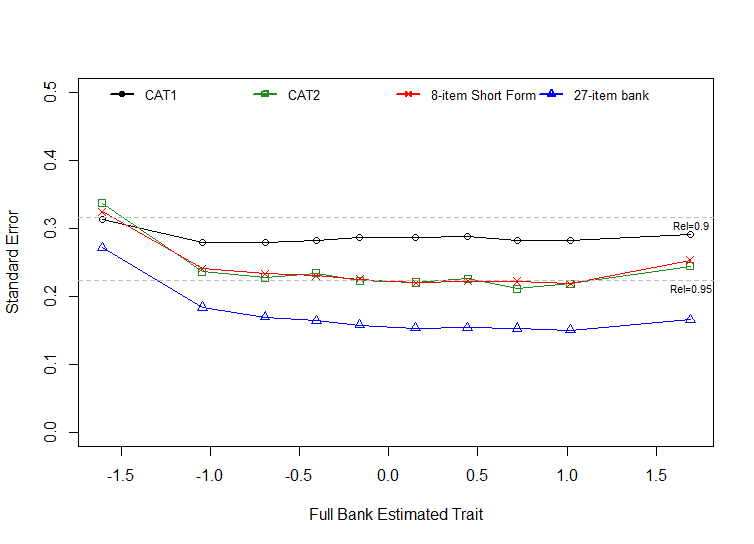


***Sensitivity Analysis – randomesque starting step***

CAT and Short Form performance versus the full bank

| **Approach** | **Correlation with full bank (Pearson)** | **RMSE** | **Mean number of items** |
| --- | --- | --- | --- |
| CAT1 (SE<0.3 or 12 items) | 0.9638 | 0.2581 | 4.53 |
| CAT2 (any 8 items) | 0.9731 | 0.2243 | 8 (fixed number) |
| PROMIS SD SF 8b | 0.9748 | 0.2166 | 8 (fixed number) |

Standard error of different approaches, split by decile of latent trait


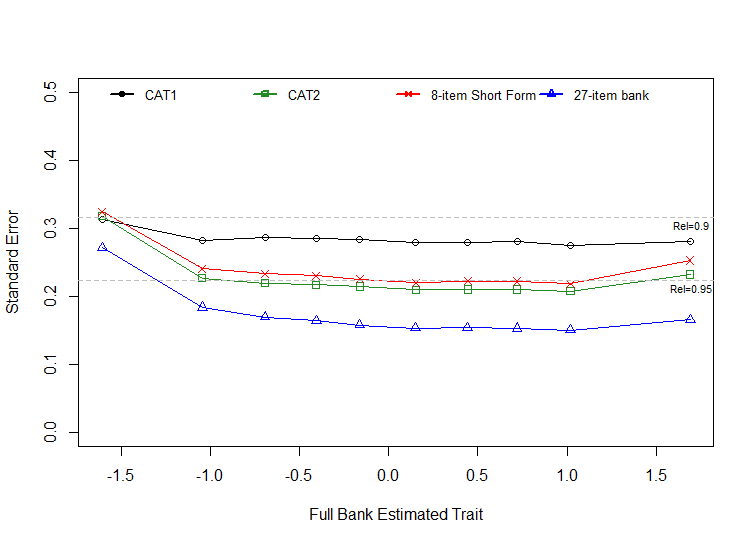


***Sensitivity Analysis – women aged 49 or above***

CAT and Short Form performance versus the full bank

| **Approach** | **Correlation with full bank (Pearson)** | **RMSE** | **Mean number of items** |
| --- | --- | --- | --- |
| CAT1 (SE<0.3 or 12 items) | 0.9621 | 0.2646 | 4.09 |
| CAT2 (any 8 items) | 0.9718 | 0.2299 | 8 (fixed number) |
| PROMIS SD SF 8b | 0.9738 | 0.2206 | 8 (fixed number) |

Standard error of different approaches, split by decile of latent trait


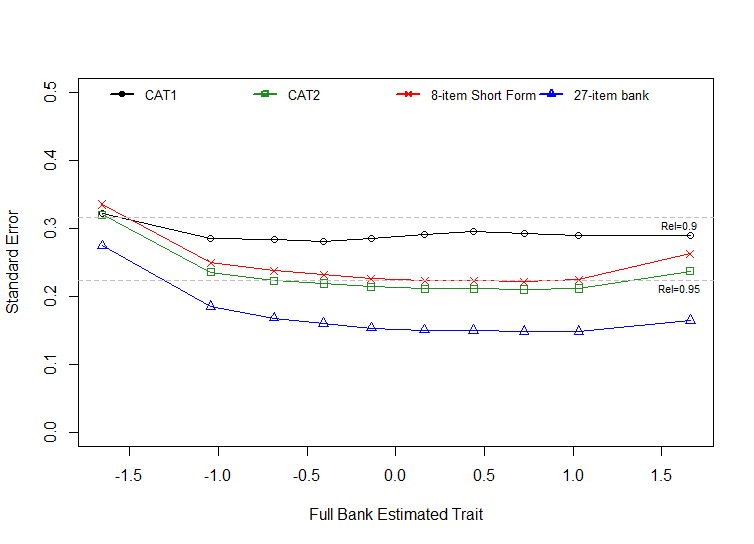


***Sensitivity Analysis – women aged 40 to 65***

CAT and Short Form performance versus the full bank

| **Approach** | **Correlation with full bank (Pearson)** | **RMSE** | **Mean number of items** |
| --- | --- | --- | --- |
| CAT1 (SE<0.3 or 12 items) | 0.9648 | 0.2563 | 3.83 |
| CAT2 (any 8 items) | 0.9845 | 0.1717 | 8 (fixed number) |
| PROMIS SD SF 8b | 0.9769 | 0.2081 | 8 (fixed number) |

Standard error of different approaches, split by decile of latent trait


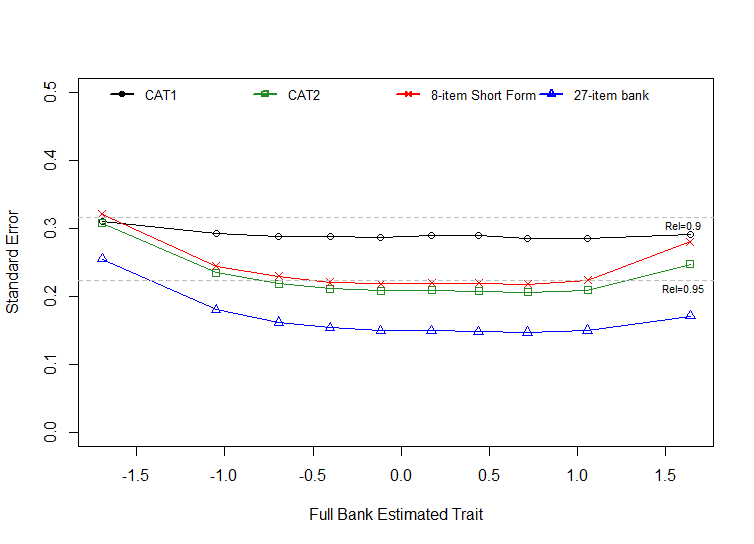


***R code***

library(mirt)

library(mirt)

library(catR)

library(dplyr)

library(haven)

library(psych)

library(DescTools)

# read data

sleepdata=read_spss("C:/.../All_SleepWake_for_SCC_022311.sav")

# filter dataset to women aged 55+

meno = sleepdata[(sleepdata$Socio02>54) & (sleepdata$Socio03==2),]

# summarise characteristics of sample

summary(as.factor(meno$Type)) # 1=general US pop, 2=general with self-reported sleep problems, 3=clinical

summary(meno$Socio02) # age

summary(as.factor(meno$Socio04)) # 1=hispanic

summary(as.factor(meno$Socio05_Asian)) # 1=Asian

summary(as.factor(meno$Socio05_Black)) # 1=Black

summary(as.factor(meno$Socio05_NativeAmericanAlaskan))

summary(as.factor(meno$Socio05_NativeHawaiianOtherPacIslander))

summary(as.factor(meno$Socio05_White))

summary(as.factor(meno$Socio07)) # education, 1-6 High School or less, 7 some college, 8 College degree, 8 advanced degree

# PSQI scoring

meno$PSQIcomp1 = ifelse(meno$PSQI14<=3,3-meno$PSQI14,NA)

meno$PSQIcomp2a = ifelse(meno$PSQI2 <= 15, 0,

ifelse(meno$PSQI2 <= 30, 1,

ifelse(meno$PSQI2 <= 60, 2,

ifelse(meno$PSQI2 <= 8000, 3, NA))))

meno$PSQIcomp2b = ifelse(meno$PSQI5 <=3, meno$PSQI5, NA)

meno$PSQIcomp2sum = meno$PSQIcomp2a + meno$PSQIcomp2b

meno$PSQIcomp2 = ifelse(meno$PSQIcomp2sum==0, 0,

ifelse(meno$PSQIcomp2sum<=2,1,

ifelse(meno$PSQIcomp2sum<=4,2,

ifelse(meno$PSQIcomp2sum<=6,3,NA))))

meno$PSQIcomp3 = ifelse(meno$PSQI4 >20, NA,

ifelse(meno$PSQI4 >7, 0,

ifelse(meno$PSQI4 >=6, 1,

ifelse(meno$PSQI4 >=5, 2, 3))))

# cannot calculate component 4 as it relies on PSQI1 and PSQI3 which have strange values - emailed query to data source but no response - do not calculate

# value of '5' could be '17:00' or '05:00'

# response to PSQI1 and PSQI3 is the same in several cases

# values such as '89'

meno$PSQIcomp5sum = meno$PSQI6 + meno$PSQI7 + meno$PSQI8 + meno$PSQI9 + meno$PSQI10 + meno$PSQI11 + meno$PSQI12 + meno$PSQI13 # no responses for 'other reasons' (PSQI Q5j)

meno$PSQIcomp5 = ifelse(meno$PSQIcomp5sum==0, 0,

ifelse(meno$PSQIcomp5sum<=9,1,

ifelse(meno$PSQIcomp5sum<=18,2,

ifelse(meno$PSQIcomp5sum<=27,3,NA))))

meno$PSQIcomp6 = meno$PSQI15

meno$PSQIcomp7sum = meno$PSQI16 + meno$PSQI17

meno$PSQIcomp7 = ifelse(meno$PSQIcomp7sum==0, 0,

ifelse(meno$PSQIcomp7sum<=2,1,

ifelse(meno$PSQIcomp7sum<=4,2,

ifelse(meno$PSQIcomp7sum<=6,3,NA))))

# ESS scoring (no skipped responses in the postmenopausal dataset)

meno$ESSscore = meno$ESS1 + meno$ESS2 + meno$ESS3 + meno$ESS4 + meno$ESS5 + meno$ESS6 + meno$ESS7 + meno$ESS8

meno$EDS = ifelse(meno$ESSscore>10,1,0)

mean(meno$EDS,na.rm=T)*100

# create dataset with the responses to the 27-item sleep disturbance bank only

meno.sd = meno %>% select(Sleep90, Sleep109, Sleep20, Sleep115, Sleep116, Sleep44, Sleep72, Sleep105, Sleep67, Sleep108, Sleep87,

Sleep45, Sleep110, Sleep92, Sleep42, Sleep78, Sleep93, Sleep125, Sleep86, Sleep68, Sleep69, Sleep65,

Sleep107, Sleep71, Sleep106, Sleep70, Sleep50)

meno.sd[meno.sd==8] = NA # recode values of 8 (skipped item) as missing

# apply reverse-scoring (the dataset codebook indicated not necessary, but I saw negative inter-item correlations/ discrimination without this step)

meno.sd.rev = meno.sd %>% mutate(Sleep109 = 4 - Sleep109,

Sleep115 = 4 - Sleep115,

Sleep116 = 4 - Sleep116,

Sleep105 = 4 - Sleep105,

Sleep110 = 4 - Sleep110,

Sleep42 = 4 - Sleep42,

Sleep107 = 4 - Sleep107)

# check for floor/ceiling effects

sumscore = meno.sd.rev %>% mutate(sum = rowSums(across(where(is.numeric))))

summary(as.factor(sumscore$sum)) # 4 people have a ceiling effect (score of zero)

# unidimensionality check

eigenvalues = eigen(polychoric(meno.sd.rev)$rho)$values

eigen.ratio = eigenvalues[1]/eigenvalues[2]

scree(polychoric(meno.sd.rev)$rho, factors=T, pc=F, hline=-1)

# fit graded response model

grm = mirt(meno.sd.rev, 1, itemtype="graded")

grmparams = coef(grm, IRTpars=T, simplify=T)$items

# local dependence check

residuals(grm, type="Q3")

# item fit check

itemfit(grm, na.rm=T, p.adjust="fdr")

# CAT post-hoc simulations

# CAT1 approach - SE<0.3 or 12 items

thetas1 = fscores(grm, method="MAP")

start1 <- list(theta = 0, startSelect = "MFI")

test1 <- list(method = "BM", itemSelect = "MFI")

stop1 <- list(rule = c("precision","length"), thr = c(0.3, 12))

final1 <- list(method = "BM")

CAT1 = simulateRespondents(thetas=thetas1, responsesMatrix=meno.sd.rev,

itemBank = grmparams, model = "GRM",

start = start1, test = test1, stop = stop1,

final = final1)

plot.catResult(CAT1,type="trueEst")

plot.catResult(CAT1,type="condBias")

plot.catResult(CAT1,type="condRMSE")

plot.catResult(CAT1,type="sError")

plot.catResult(CAT1,type="numberItems")

plot.catResult(CAT1,type="cumNumberItems")

CAT1full=CAT1$final.values.df

CAT1full$prop4 = ifelse(CAT1full$total.items.administrated<=4,1,0)

mean(CAT1full$prop4)*100 # proportion of times 4 or less items are administered

CAT1$exposureRates # proportion each item is shown

# CAT2 approach - any 8 items

thetas2 = fscores(grm, method="MAP")

start2 <- list(theta = 0, startSelect = "MFI")

test2 <- list(method = "BM", itemSelect = "MFI")

stop2 <- list(rule = "length", thr = 8)

final2 <- list(method = "BM")

CAT2 = simulateRespondents(thetas=thetas2, responsesMatrix=meno.sd.rev,

itemBank = grmparams, model = "GRM",

start = start2, test = test2, stop = stop2,

final = final2)

plot.catResult(CAT2,type="trueEst")

plot.catResult(CAT2,type="condBias")

plot.catResult(CAT2,type="condRMSE")

plot.catResult(CAT2,type="sError")

CAT2full=CAT2$final.values.df

CAT2$exposureRates # proportion each item is shown

# Fix the items of the 8 item short form (PROMIS SD SF 8b)

thetas3 = fscores(grm, method="MAP")

start3 <- list(fixItems=c(1,2,4,5,6,10,11,13))

test3 <- list(method = "BM", itemSelect = "MFI")

stop3 <- list(rule = "length", thr = 8)

final3 <- list(method = "BM")

SF = simulateRespondents(thetas=thetas3, responsesMatrix=meno.sd.rev,

itemBank = grmparams, model = "GRM",

start = start3, test = test3, stop = stop3,

final = final3)

plot.catResult(SF,type="trueEst")

plot.catResult(SF,type="condBias")

plot.catResult(SF,type="condRMSE")

plot.catResult(SF,type="sError")

SFfull=SF$final.values.df

# All 27 items in the bank - to get comparable standard errors

thetas4 = fscores(grm, method="MAP")

start4 <- list(theta = 0, startSelect = "MFI")

test4 <- list(method = "BM", itemSelect = "MFI")

stop4 <- list(rule = "length", thr = 27)

final4 <- list(method = "BM")

bank = simulateRespondents(thetas=thetas4, responsesMatrix=meno.sd.rev,

itemBank = grmparams, model = "GRM",

start = start4, test = test4, stop = stop4,

final = final4)

plot.catResult(bank,type="sError")

bankfull=bank$final.values.df

# comparison plot of SEs including reliability

plot(CAT1$condTheta, CAT1$condSE, type="o",

xlab="Full Bank Estimated Trait", ylab="Standard Error",

ylim=c(0,0.5))

lines(CAT2$condTheta, CAT2$condSE, type="o", col="forestgreen", pch=0)

lines(SF$condTheta, SF$condSE, type="o", col="red", pch=4)

lines(bank$condTheta, bank$condSE, type="o", col="blue", pch=2)

legend("top", legend = c("CAT1","CAT2","8-item Short Form", "27-item bank"),

lwd = 2, col = c("black","forestgreen", "red","blue"), pch=c(1,0,4,2), cex=0.8, horiz=T, bty = "n")

abline(h=0.316,lty=2,col="gray") # technically SE=0.3162 corresponds to Rel=0.9 (information=9, Bayes Modal)

text("Rel=0.9",x=1.7,y=0.305, cex=0.7)

abline(h=0.224,lty=2,col="gray") # technically SE=0.2236 corresponds to Rel=0.95 (information=19, Bayes Modal)

text("Rel=0.95",x=1.7,y=0.213, cex=0.7)

# relative efficiency of different algorithms (efficiency = information / n_items)

RE = cbind.data.frame(CAT1$condTheta, CAT1$condSE, CAT1$condnItems,

CAT2$condTheta, CAT2$condSE, CAT2$condnItems,

SF$condTheta, SF$condSE, SF$condnItems,

bank$condTheta, bank$condSE, bank$condnItems)

RE$CAT1info = (1/CAT1$condSE^2)-1

RE$CAT1efficiency = RE$CAT1info / CAT1$condnItems

RE$CAT2info = (1/CAT2$condSE^2)-1

RE$CAT2efficiency = RE$CAT2info / CAT2$condnItems

RE$SFinfo = (1/SF$condSE^2)-1

RE$SFefficiency = RE$SFinfo / SF$condnItems

RE$bankinfo = (1/bank$condSE^2)-1

RE$bankefficiency = RE$bankinfo / bank$condnItems

RE$CAT1vSF = RE$CAT1efficiency / RE$SFefficiency

RE$CAT2vSF = RE$CAT2efficiency / RE$SFefficiency

RE$CAT1vCAT2 = RE$CAT1efficiency / RE$CAT2efficiency

plot(CAT1$condTheta, RE$CAT1vSF, type="o",

xlab="Full Bank Estimated Trait", ylab="Relative Efficiency",

ylim=c(0,2))

lines(CAT2$condTheta, RE$CAT2vSF, type="o", col="orange")

lines(SF$condTheta, RE$CAT1vCAT2, type="o", col="blue")

legend("top", legend = c("CAT1 vs SF","CAT2 vs SF","CAT1 vs CAT2"),

lwd = 2, col = c("black","orange","blue"), cex=0.8, horiz=T, bty = "n")

abline(h=1,lty=2,col="gray")

# correlation between PROMIS, PSQI components (polyserial), ESS (Spearman)

# ESS

SpearmanRho(CAT1full$estimated.theta, meno$ESSscore,use="complete.obs", conf.level=0.95)

SpearmanRho(CAT2full$estimated.theta, meno$ESSscore,use="complete.obs", conf.level=0.95)

SpearmanRho(SFfull$estimated.theta, meno$ESSscore,use="complete.obs", conf.level=0.95)

SpearmanRho(bankfull$estimated.theta, meno$ESSscore,use="complete.obs", conf.level=0.95)

# PSQI component 1 - Subjective sleep quality

polyserial(as.matrix(CAT1full$estimated.theta), as.matrix(meno$PSQIcomp1))

CorCI(polyserial(as.matrix(CAT1full$estimated.theta), as.matrix(meno$PSQIcomp1)),n=nrow(meno.sd.rev))

polyserial(as.matrix(CAT2full$estimated.theta), as.matrix(meno$PSQIcomp1))

CorCI(polyserial(as.matrix(CAT2full$estimated.theta), as.matrix(meno$PSQIcomp1)),n=nrow(meno.sd.rev))

polyserial(as.matrix(SFfull$estimated.theta), as.matrix(meno$PSQIcomp1))

CorCI(polyserial(as.matrix(SFfull$estimated.theta), as.matrix(meno$PSQIcomp1)),n=nrow(meno.sd.rev))

polyserial(as.matrix(bankfull$estimated.theta), as.matrix(meno$PSQIcomp1))

CorCI(polyserial(as.matrix(bankfull$estimated.theta), as.matrix(meno$PSQIcomp1)),n=nrow(meno.sd.rev))

# PSQI component 2 - Sleep latency

polyserial(as.matrix(CAT1full$estimated.theta), as.matrix(meno$PSQIcomp2))

CorCI(polyserial(as.matrix(CAT1full$estimated.theta), as.matrix(meno$PSQIcomp2)),n=nrow(meno.sd.rev))

polyserial(as.matrix(CAT2full$estimated.theta), as.matrix(meno$PSQIcomp2))

CorCI(polyserial(as.matrix(CAT2full$estimated.theta), as.matrix(meno$PSQIcomp2)),n=nrow(meno.sd.rev))

polyserial(as.matrix(SFfull$estimated.theta), as.matrix(meno$PSQIcomp2))

CorCI(polyserial(as.matrix(SFfull$estimated.theta), as.matrix(meno$PSQIcomp2)),n=nrow(meno.sd.rev))

polyserial(as.matrix(bankfull$estimated.theta), as.matrix(meno$PSQIcomp2))

CorCI(polyserial(as.matrix(bankfull$estimated.theta), as.matrix(meno$PSQIcomp2)),n=nrow(meno.sd.rev))

# PSQI component 3 - Sleep duration

polyserial(as.matrix(CAT1full$estimated.theta), as.matrix(meno$PSQIcomp3))

CorCI(polyserial(as.matrix(CAT1full$estimated.theta), as.matrix(meno$PSQIcomp3)),n=nrow(meno.sd.rev))

polyserial(as.matrix(CAT2full$estimated.theta), as.matrix(meno$PSQIcomp3))

CorCI(polyserial(as.matrix(CAT2full$estimated.theta), as.matrix(meno$PSQIcomp3)),n=nrow(meno.sd.rev))

polyserial(as.matrix(SFfull$estimated.theta), as.matrix(meno$PSQIcomp3))

CorCI(polyserial(as.matrix(SFfull$estimated.theta), as.matrix(meno$PSQIcomp3)),n=nrow(meno.sd.rev))

polyserial(as.matrix(bankfull$estimated.theta), as.matrix(meno$PSQIcomp3))

CorCI(polyserial(as.matrix(bankfull$estimated.theta), as.matrix(meno$PSQIcomp3)),n=nrow(meno.sd.rev))

# PSQI component 5 - Sleep disturbances

polyserial(as.matrix(CAT1full$estimated.theta), as.matrix(meno$PSQIcomp5))

CorCI(polyserial(as.matrix(CAT1full$estimated.theta), as.matrix(meno$PSQIcomp5)),n=nrow(meno.sd.rev))

polyserial(as.matrix(CAT2full$estimated.theta), as.matrix(meno$PSQIcomp5))

CorCI(polyserial(as.matrix(CAT2full$estimated.theta), as.matrix(meno$PSQIcomp5)),n=nrow(meno.sd.rev))

polyserial(as.matrix(SFfull$estimated.theta), as.matrix(meno$PSQIcomp5))

CorCI(polyserial(as.matrix(SFfull$estimated.theta), as.matrix(meno$PSQIcomp5)),n=nrow(meno.sd.rev))

polyserial(as.matrix(bankfull$estimated.theta), as.matrix(meno$PSQIcomp5))

CorCI(polyserial(as.matrix(bankfull$estimated.theta), as.matrix(meno$PSQIcomp5)),n=nrow(meno.sd.rev))

# PSQI component 6 - Use of sleeping medication

polyserial(as.matrix(CAT1full$estimated.theta), as.matrix(meno$PSQIcomp6))

CorCI(polyserial(as.matrix(CAT1full$estimated.theta), as.matrix(meno$PSQIcomp6)),n=nrow(meno.sd.rev))

polyserial(as.matrix(CAT2full$estimated.theta), as.matrix(meno$PSQIcomp6))

CorCI(polyserial(as.matrix(CAT2full$estimated.theta), as.matrix(meno$PSQIcomp6)),n=nrow(meno.sd.rev))

polyserial(as.matrix(SFfull$estimated.theta), as.matrix(meno$PSQIcomp6))

CorCI(polyserial(as.matrix(SFfull$estimated.theta), as.matrix(meno$PSQIcomp6)),n=nrow(meno.sd.rev))

polyserial(as.matrix(bankfull$estimated.theta), as.matrix(meno$PSQIcomp6))

CorCI(polyserial(as.matrix(bankfull$estimated.theta), as.matrix(meno$PSQIcomp6)),n=nrow(meno.sd.rev))

# PSQI component 7 - Daytime dysfunction

polyserial(as.matrix(CAT1full$estimated.theta), as.matrix(meno$PSQIcomp7))

CorCI(polyserial(as.matrix(CAT1full$estimated.theta), as.matrix(meno$PSQIcomp7)),n=nrow(meno.sd.rev))

polyserial(as.matrix(CAT2full$estimated.theta), as.matrix(meno$PSQIcomp7))

CorCI(polyserial(as.matrix(CAT2full$estimated.theta), as.matrix(meno$PSQIcomp7)),n=nrow(meno.sd.rev))

polyserial(as.matrix(SFfull$estimated.theta), as.matrix(meno$PSQIcomp7))

CorCI(polyserial(as.matrix(SFfull$estimated.theta), as.matrix(meno$PSQIcomp7)),n=nrow(meno.sd.rev))

polyserial(as.matrix(bankfull$estimated.theta), as.matrix(meno$PSQIcomp7))

CorCI(polyserial(as.matrix(bankfull$estimated.theta), as.matrix(meno$PSQIcomp7)),n=nrow(meno.sd.rev))

# known-groups comparisons

meno$reportproblems = ifelse(meno$Type>1, 1, 0)

t.test(CAT1full$estimated.theta ~ meno$reportproblems)

cohen.d(as.data.frame(cbind(CAT1full$estimated.theta,meno$reportproblems)),"V2")

t.test(CAT2full$estimated.theta ~ meno$reportproblems)

cohen.d(as.data.frame(cbind(CAT2full$estimated.theta,meno$reportproblems)),"V2")

t.test(SFfull$estimated.theta ~ meno$reportproblems)

cohen.d(as.data.frame(cbind(SFfull$estimated.theta,meno$reportproblems)),"V2")

t.test(bankfull$estimated.theta ~ meno$reportproblems)

cohen.d(as.data.frame(cbind(bankfull$estimated.theta,meno$reportproblems)),"V2")
